# Unhealthy and Unequal: Socioeconomic Vulnerability Shapes Dietary Quality in Children and Adolescents from Spain

**DOI:** 10.3390/nu17233635

**Published:** 2025-11-21

**Authors:** María González-Rodríguez, Julia Almazán-Catalán, Marina Redruello-Requejo, Carmen Morais-Moreno, Alejandra Carretero-Krug, Ana M. Puga, Ana Montero-Bravo, María de Lourdes Samaniego-Vaesken, Teresa Partearroyo, Gregorio Varela-Moreiras

**Affiliations:** 1 Grupo USP-CEU de Excelencia “Nutrición para la Vida (Nutrition for Life)”, Ref: E02/0720, Departamento de Ciencias Farmacéuticas y de la Salud, Facultad de Farmacia, Universidad San Pablo-CEU, CEU Universities, Urbanización Montepríncipe, 28660 Madrid, Spain; m.gonzalez202@usp.ceu.es (M.G.-R.); julia.almazancatalan@ceu.es (J.A.-C.); m.redruello@usp.ceu.es (M.R.-R.); carmen.moraismoreno@ceu.es (C.M.-M.); alejandra.carreterokrug@ceu.es (A.C.-K.); anamaria.pugagimenezazca@ceu.es (A.M.P.); amontero.fcex@ceu.es (A.M.-B.); l.samaniego@ceu.es (M.d.L.S.-V.); gvarela@ceu.es (G.V.-M.); 2 Instituto Universitario CEU Alimentación y Sociedad, Facultad de Farmacia, Universidad San Pablo-CEU, CEU Universities, Urbanización Montepríncipe, Boadilla del Monte, 28660 Madrid, Spain

**Keywords:** socioeconomic vulnerability, diet quality, Mediterranean diet, ultra-processed foods, childhood obesity, malnutrition, Spain

## Abstract

**Background/Objectives**: Childhood obesity and nutritional inequalities remain major public health challenges, particularly in socioeconomically disadvantaged settings. In Spain, these disparities are reflected in unequal access to healthy food and differing health outcomes among the young population. This study aimed to explore how social vulnerability influences dietary patterns, body composition, and food insecurity among children and adolescents, with a particular focus on sex differences. **Methods**: A descriptive cross-sectional study was conducted with 280 participants aged 6 to 15 years old, recruited from urban areas across Spain. Two groups were evaluated, a socioeconomically vulnerable group (VG) (*n* = 175) and a non-vulnerable group (NVG) (*n* = 105), classified according to socioeconomic and social established criteria. Validated tools were used to assess diet quality, adherence to the Mediterranean Diet (MD), consumption of ultra-processed foods (UPF), and household food insecurity. Anthropometric measurements were also collected, and body mass index (BMI) was calculated using both national and international reference standards. **Results**: Vulnerable children and adolescents showed higher prevalence of food insecurity, less favorable body composition indicators, and lower global dietary quality, characterized by lower adherence to the MD and higher consumption of UPF. Multivariate analysis confirmed that socioeconomic vulnerability was significantly associated with female sex, higher BMI, lower adherence to the MD, and greater consumption of UPF. **Conclusions**: Our findings highlight a concerning pattern of health and nutritional inequality among children and adolescents based on socioeconomic status. There is a clear and urgent need for effective public health strategies with an equity focus that promote healthy and affordable eating habits from early life, especially in the most disadvantaged environments and targeted by gender.

## 1. Introduction

Contemporary life has been shaped by rapid urbanization, population growth, technological progress, and global challenges such as climate change and geopolitical tensions, all of which have disrupted food systems and altered food production, distribution, and consumption patterns. In recent decades, the number of people experiencing hunger has increased due to the intensification of key factors of food insecurity and malnutrition. Furthermore, persistent social inequalities and various forms of child malnutrition, such as stunting, micronutrient deficiencies, and rising rates of overweight and obesity, continue to pose major public health challenges [1].

The 2024 State of Food Security and Nutrition in the World (SOFI) report highlighted that progress toward Sustainable Development Goal 2 (Zero Hunger) has stagnated, with 733 million people undernourished in 2023 [2]. Food insecurity, defined as limited or uncertain access to nutritionally adequate and safe food, affected 28.9% of the global population (approximately 2.33 billion people), impairing child development and academic outcomes [3]. Additionally, it was estimated that in 2022, over 2.8 billion people could not afford a healthy diet, underscoring the growing gap between food availability and accessibility [2].

One of the most concerning consequences of nutritional inequality is the so-called “hidden hunger”, a form of micronutrient deficiency that results from poor dietary diversity. This condition affects both undernourished populations and those consuming excessive calories from nutrient-poor foods [4]. Certainly, poverty remains a major barrier to accessing balanced and diverse diets [5], particularly among socially vulnerable groups that also face health, educational, and economic disadvantages [6].

In parallel, the global rise of overweight and obesity, driven by the nutrition transition, has become a pressing issue [7]. Childhood obesity has surged especially over the past two decades, with long-term consequences such as type 2 diabetes, cardiovascular disease, and early mortality [8]. Excess weight from an early age represents a serious public health concern, as children with obesity are approximately five times more likely to remain obese in adulthood, thereby perpetuating intergenerational cycles of poor health [9].

Diet quality is a key determinant of both weight management and overall health. In this context, the Mediterranean Diet (MD) has been consistently associated with positive health outcomes and effective weight regulation [9,10], emphasizing the importance of promoting healthy dietary patterns from early life to reduce long-term health risks.

However, adherence to healthy dietary patterns is increasingly threatened by the growing consumption of ultra-processed foods (UPFs), which has become one of the most influential dietary factors shaping nutritional quality. These products, typically high in added sugars, saturated fats, and sodium, displace healthier dietary patterns such as the MD, especially among children and adolescents [11,12,13,14,15] and are strongly associated with obesity risk [16,17,18] as well as with hidden hunger. Their high palatability and commonly low nutritional value contribute to excessive energy intake, undermining efforts to foster healthy eating behaviors [19,20,21].

Beyond dietary habits, a range of sociodemographic factors also play a crucial role in shaping nutritional status and obesity risk. Sociodemographic determinants such as sex, age, socioeconomic status, and migrant background are also closely associated with excessive weight in children and adolescents [22]. Spain reflects somewhat this global trend. In fact, the ALADINO 2023 study reports that more than one-third of children aged 6–9 are affected by excessive weight, particularly in socioeconomically disadvantaged households [23]. Adolescence is a particularly sensitive period marked by physiological, behavioral, and psychological changes that heighten susceptibility to weight gain [24,25].

Finally, national reports from Spanish non-governmental organizations (NGOs) and institutions such as FOESSA and Cáritas emphasize that dietary improvements have not reached the most vulnerable populations, highlighting the persistent social gradient in nutritional health [26]. These organizations document how socioeconomic hardship continues to limit access to balanced diets, particularly among households with children, single-parent families, and migrant populations. Such evidence points to a widening gap in nutritional well-being, requiring comprehensive public health strategies that integrate food security, education, and equity considerations.

This study aims to investigate differences and similarities in dietary patterns, quality and body composition between socioeconomically vulnerable and non-vulnerable Spanish children and adolescents, with a specific focus on sex-based disparities and their implications for designing targeted public health interventions and policies to reduce nutritional inequalities.

## 2. Materials and Methods

### 2.1. Study Design

A descriptive, cross-sectional observational study was conducted involving school-aged children and adolescents between 6 and 15 years old (from primary education to the final year of secondary education). The study was carried out between 2022 and 2025. Participants were selected according to predefined inclusion and exclusion criteria. Individuals were excluded if they fell outside the defined age range, if informed consent was not obtained from parents or legal guardians, or if difficulties arose in understanding or completing questionnaires.

Participants could withdraw at any time by revoking consent. In addition, the research team could also exclude participants who did not meet inclusion criteria, development of health conditions affecting dietary patterns, or provided incomplete questionnaire data.

### 2.2. Study Participants and Sample Size

A convenience sampling strategy yielded 280 participants from metropolitan areas across Spain. Data collection was conducted in Andalusia (Seville), Galicia (Ferrol), the Community of Madrid (Moratalaz, Usera, and Villaverde), and Melilla for the socioeconomically vulnerable group, and in Madrid for the non-vulnerable group.

The sample included a socioeconomically vulnerable cohort (*n* = 175) and a non-vulnerable control group (*n* = 105). Participants were considered vulnerable based on criteria such as family inclusion in social integration programs, receipt of food assistance from food banks, and/or parental employment status.

Eligible Participants were school-aged children or adolescents (6–15 years), regularly attending school, and able to complete the questionnaires with parental assistance when required.

After informed consent was obtained, participants completed the questionnaires and underwent standardized anthropometric assessment.

### 2.3. Ethical Considerations

Ethical approval was granted by the Clinical Research Ethics Committee of CEU San Pablo University (ethical code 601/22/58). The study complied with the Declaration of Helsinki and subsequent amendments. Participants and/or guardians received detailed study information and signed informed consent before participation. All data were handled confidentially according to General Data Protection Regulation 2016/679 [27] and the Organic Law 3/2018 for the Protection of Personal Data and the guarantee of digital rights [28].

### 2.4. Dietary and Sociodemographic Indices

To comprehensively evaluate the relationship between social vulnerability and nutritional outcomes, the following validated instruments were administered by qualified personnel with training in human nutrition and dietetics:

▪ Sociodemographic and Health Questionnaire: A structured questionnaire used to collect information on participant’s age, sex, socioeconomic status, parental education level, household composition, vulnerability, as well as general health status, presence of chronic or acute diseases, recent dietary modifications due to illness, allergies or surgical interventions, and other relevant health-related factors.
▪ Household Food Insecurity Access Scale (HFIAS): A validated tool developed by the Food and Nutrition Technical Assistance III (FANTA) [29] to assess the degree of food insecurity experienced by households over the previous 30 days. It measures access-related dimensions of food insecurity through perceptions and behavioral responses to limited food access.
▪ Spanish Healthy Eating Index (IASE): this index provides an integrated measure of overall dietary quality. It is adapted from the American Healthy Eating Index [30] and is tailored to the Spanish population [31]. It is based on the adherence to national dietary guidelines and evaluates the frequency and adequacy of consumption across various food groups and nutrients [32]. The total score, based on a maximum of 100 points, classified diets into three categories: “healthy” (>80 points), “needs improvement” (51–80), and “unhealthy” (≤50).
▪ MD Adherence (KIDMED): The KIDMED index [33] evaluates adherence to the MD in children and adolescents. It consists of 16 items (12 positive and 4 negative) that assess the frequency of consumption of key food groups aligned with Mediterranean dietary patterns. Participants were classified into the three predefined categories [33] based on their overall score: (1) >8, High adherence to MD; (2) 4–7, moderate adherence to MD; (3) <3, Low adherence to MD.
▪ UPF Intake (sQ-HPF): The short questionnaire to assess the intake of HPF (Highly Processed Foods), sQ-HPF, quantifies the frequency and extent of consumption of foods classified as UPF according to the NOVA classification. It provides a simple and effective tool to evaluate dietary quality and the degree of food processing [34,35]. Participants were classified into three categories of UPF intake, where the proportion of UPF consumption relative to total daily intake (in grams) was estimated and divided into tertiles: low (11.3–22.4%), moderate (26.1–44.6%), and high (48.3–59.4%).

### 2.5. Anthropometric Assessment

Anthropometric assessment included weight measurement using a Tanita^TM^ Body Composition Analyzer (Tanita Corporation, Tokyo, Japan), and height measurement using a SECA^TM^ portable stadiometer (model 123) (SECA GmbH & Co. KG, Hamburg, Germany). All anthropometric measurements were conducted in accordance with the recommendations of the International Standards for Anthropometric Assessment (ISAK) [36] by certified Level 1 and 2 anthropometrists. Body weight (kg) and height (cm) data were used to calculate the Body Mass Index (BMI), which allowed participants to be classified into categories of severe thinness, underweight, normal weight, overweight, or obesity based on percentile cut-offs established by the Faustino Orbegozo Foundation (OF) [37]. In addition, the World Health Organization (WHO) reference standards were used to calculate BMI-for-age Z-scores [38,39] according to sex and age.

### 2.6. Statistical Analysis

Data analysis was carried out using IBM SPSS Statistics version 29.0. Descriptive statistics were used to summarize the main characteristics of the sample. Continuous variables were presented as medians and interquartile ranges (IQR), as most did not follow a normal distribution (confirmed by the Kolmogorov–Smirnov test). Categorical variables were expressed as absolute and relative frequencies (*n*, %).

To explore differences between vulnerable and non-vulnerable participants, the Mann–Whitney *U* test for continuous variables and the Fisher–Freeman–Halton exact test for categorical variables were used. Analyses were also stratified by sex to examine potential differences; *p*-values below 0.05 were considered as statistically significant.

Additionally, a binary logistic regression was performed to identify which variables were associated with vulnerability status. The model included sex, age, BMI, adherence to the MD (KIDMED), and level of UPF consumption as independent variables. Odds ratios (OR) and 95% confidence intervals (CI) were calculated. The model’s explanatory capacity was assessed using Cox and Snell and Nagelkerke R^2^ values, and overall fit was checked with the chi-square test.

## 3. Results

### 3.1. General Description

The analyzed population comprised a total of 280 participants aged between 6 and 15 years, of whom 175 were identified as socially vulnerable and 105 as non-vulnerable. Median age was comparable between groups, showing no statistically significant differences. Median age in the non-vulnerable group (NVG) was 9 years (7.2–11.0), and in the vulnerable group (VG) 9 years (8.0–11.0).

A significant association was found between disease presence and vulnerability status (*p* ≤ 0.05). In the VG, 19.4% of participants reported having a disease, compared with 7.6% in the NVG. Overall, 85.0% of the total sample reported no disease and 15.0% reported the presence of disease.

Parental employment status also differed significantly between groups (*p* ≤ 0.05). In the VG, 42.9% of participants lived in households where neither parent was employed, compared with 3.9% in the NVG. Overall, 93.4% of fathers and 76.2% of mothers were employed. Among the four non-vulnerable participants whose parents were both classified as not working, one case corresponded to a retired father and three to mothers identified as homemakers.

### 3.2. Food Insecurity

Table 1 presents the classification of children and adolescents by vulnerability status and sex according to household food insecurity levels (HFIAS) [29].

Within the vulnerable population, approximately three out of four participants experienced some degree of food insecurity, distributed as mild (22.9%), moderate (23.4%), and severe (28.6%), while 100% of the NVG were food secure. The differences between groups were statistically significant (*p* ≤ 0.001).

When disaggregated by sex, similar patterns were observed among vulnerable boys and girls, both showing significantly higher rates of food insecurity compared with the non-vulnerable group, which was uniformly food secure (*p* ≤ 0.001).

### 3.3. Body Composition

Anthropometric results are shown in Table 2. Significant differences were observed between groups. Vulnerable participants presented higher BMI values and higher BMI-for-age Z-scores compared with the non-vulnerable group (*p* ≤ 0.05). Height-for-age Z-scores showed no statistically significant differences.

When stratifying by sex, girls in the VG showed higher BMI and BMI-for-age Z-scores compared with girls in the NVG (*p* ≤ 0.05), while differences among boys followed the same direction but did not reach significance.

Table 3 displays overweight and obesity prevalence according to the OF and WHO reference standards [37,38,39]. According to the OF criteria [37], the VG showed significantly higher prevalence rates of overweight (33.1%) and obesity (10.9%), compared to the NVG, where only 9.5% were classified as overweight with no cases of obesity were observed. In contrast, the NVG had higher proportions of severe thinness and underweight (*p* ≤ 0.001).

When using the WHO standards [38,39], obesity was markedly more frequent in the VG (37.7%) compared with the NVG (6.7%) (*p* ≤ 0.001).

Stratified by both vulnerable boys and girls exhibited higher overweight and obesity rates than their non-vulnerable counterparts (*p* ≤ 0.001).

### 3.4. Diet Quality

Diet quality indicators are summarized in Table 4. Vulnerable participants obtained lower IASE scores than their non-vulnerable peers (*p* ≤ 0.001). Most children in the VG fell into the “needs improvement” or “unhealthy” diet categories, while a larger proportion of NVG participants achieved “healthy” diet scores.

KIDMED scores also differed significantly. Vulnerable participants showed lower adherence to the Mediterranean Diet, with a higher proportion falling into the low-adherence category (*p* ≤ 0.001).

UPF consumption patterns further highlighted nutritional disparities. The VG exhibited higher ultra-processed food intake, with more participants falling into the moderate and high tertiles compared with the NVG (*p* ≤ 0.001).

### 3.5. Sociodemographic and Dietary Factors Associated with Vulnerability

Table 5 displays the results of the binary logistic regression analysis assessing factors associated with vulnerability. The model showed strong explanatory power (Cox and Snell R^2^ = 0.317; Nagelkerke R^2^ = 0.433; *p* < 0.001).

Sex, BMI, adherence to the MD, and UPF intake were significantly associated with vulnerability. Girls were less likely to be classified as vulnerable (OR = 0.37, *p* < 0.01), whereas higher BMI values increased the likelihood of vulnerability (OR = 1.41, *p* < 0.001). Participants with moderate adherence to the MD were three times more likely to be vulnerable (OR = 3.18, *p* < 0.001), while those with low adherence showed a tenfold higher risk (OR = 10.27, *p* < 0.001) compared to those with high adherence.

Similarly, UPF intake was strongly associated with vulnerability, as compared to low consumers, medium consumption increased the odds threefold (OR = 3.18, *p* < 0.01) and high consumption more than twentyfold (OR = 24.57, *p* < 0.01).

## 4. Discussion

The present study provides original evidence on the relationship between socioeconomic vulnerability, dietary quality, and body composition among Spanish children and adolescents. Our findings show clear disparities between vulnerable and non-vulnerable groups, with the former presenting poorer diet quality, lower adherence to the Mediterranean diet, higher ultra-processed food consumption, and greater prevalence of overweight and obesity. These results highlight the nutritional consequences of social inequality and emphasize the need for targeted public health strategies addressing food insecurity and unhealthy dietary habits.

These findings align with those reported in larger population studies, which consistently describe similar patterns of dietary and nutritional disparities among socioeconomically disadvantaged children and adolescents. Although the limited sample size constrains the generalizability of our results, the trends observed are in line with national evidence indicating persistent disparities in diet and nutrition across Spain. Our findings suggest the existence of dietary and nutritional disparities among socioeconomically disadvantaged Spanish children and adolescents, compared with their non-vulnerable peers, revealing patterns consistent with social inequalities reported in larger population studies. This overall consistency reinforces the relevance of our observations within the broader national context.

Recent national reports contextualize these findings. In 2023, the percentage of the Spanish population at risk of poverty or social exclusion in Spain rose to 26.5%, up from 26.0% in 2022 [40]. According to the 2022 Living Conditions Survey conducted by the National Institute of Statistics (INE) [41], 5.4% of Spanish households could not afford a meal containing meat, chicken, or fish (or vegetarian equivalents) at least every two days. This corresponds to more than one million households facing food-related hardships [26]. Even more concerning is that this percentage has steadily increased since records from 2004, when it stood at 2.3%, reflecting a doubling of households forced to reduce animal protein intake in recent years. Protein consumption serves as a key indicator of material and social deprivation in Spanish households, as access to protein-rich foods is closely tied to quality of life and nutritional well-being [26], especially among growing populations (children and adolescents).

Specifically, in our study, overweight and obesity prevalence reached 33.1% and 10.9% in the vulnerable group, while no cases of obesity were recorded among the non-vulnerable group. Similarly, adherence to the Mediterranean diet was notably lower among vulnerable participants (29.7% low adherence) compared with their non-vulnerable peers (41.0% high adherence). These results are consistent with national surveys such as ALADINO 2023 [23] and PASOS 2022–2023 [42], which also report higher rates of excess weight and lower diet quality in children from lower socioeconomic backgrounds.

These patterns of material deprivation are closely linked to social vulnerability factors that contribute to food insecurity among children. A recent systematic review conducted in high-income countries identified low household income as the most common determinant of food insecurity. Additional factors highlighted included the child’s age, parental depression, household overcrowding, ethnicity and experiences of racism, psychosocial and physical health status, social isolation, and residential instability [43].

Consistent with this evidence, studies by the FOESSA Foundation [44] reported in 2021 that 2.8% of the Spanish population experienced hunger or had frequently experienced it in recent years, equivalent to 2.6% of all households. Furthermore, 11.4% of households could not afford an adequate diet according to sex and age. These findings are consistent with the “Study on Food Insecurity in Spanish Households Before and During COVID-19” conducted by the University of Barcelona. Using the United Nations Food Insecurity Experience Scale (FIES) [45], the study showed that food insecurity in Spain rose from 11.9% to 13.3% after the pandemic. Importantly, this highlights that food insecurity is a structural issue that predated COVID-19 and is not merely a temporary phenomenon. Notably, nearly 2.5 million individuals have been forced to reduce their daily food intake due to a lack of economic resources, experiencing severe food insecurity—twice the figure recorded at the start of the pandemic [46]. Together, these indicators reinforce the structural nature of food inequalities in Spain.

These social and nutritional inequalities are especially pronounced among children, where the link between socioeconomic disadvantage and excess weight is well established [47,48,49,50,51]. In developed countries, childhood inequality is commonly associated with excess weight. Indeed, Spain ranks among European Union (EU) countries with the highest associations between child poverty and obesity [52,53]. At the household level, obesity prevalence among children is more than twice as high in low-income families compared to those from higher-income households [53]. Both overweight and obesity are more frequent in low-resource households and tend to decrease as household income increases [6].

In our analysis stratified by sex, obesity rates were higher among girls than boys, particularly within the vulnerable group, underscoring the need for gender-sensitive approaches when designing prevention strategies.

In addition, scientific evidence supports the effectiveness of community-based interventions in preventing childhood obesity across diverse socioeconomic contexts [54,55]. These programs are particularly important for children from vulnerable or low-income families, who are at higher risk of excess weight, as demonstrated in recent studies [54]. Thus, health programs focusing on children and adolescents are especially critical in the context of ongoing economic instability.

In this regard, since 2017, the WHO has considered childhood obesity one of the most serious public health challenges of the 21st century [56]. Children with obesity are more likely to become obese adults and to face an earlier onset of conditions such as diabetes and cardiovascular disease, compared to their peers of healthy weight [56]. In addition, scientific evidence [57,58,59] has shown significant discrepancies in the diagnosis of childhood overweight and obesity depending on the growth reference used, as each system applies different cut-off points. According to the latest Spanish National Strategic Plan against Childhood Obesity [53], four out of ten children in Spain are affected by excess weight, and it is estimated that 55% of children with obesity will become adolescents with this condition. In turn, 80% of those adolescents are expected to become adults with obesity. These data underscore that childhood obesity represents one of the most pressing public health concerns due to its physical, psychological, and social consequences throughout the life course [53]. To further contextualize our findings, BMI classification in this study was performed using both WHO and OF criteria, which represent international and Spanish references, respectively, and yielded comparable trends to those observed in national surveillance programs [60,61].

The latest ALADINO 2023 study [23] confirms these disparities. Socioeconomic analysis revealed a higher prevalence of excess weight in low- and middle-income households, with minimal improvement over previous survey periods in the lowest-income group. Our results align with these national data, showing consistently higher rates of overweight and obesity among children in the vulnerable group compared with their non-vulnerable peers, using both WHO and OF standards. The ALADINO 2023 study further linked higher excess weight prevalence to factors such as parental unemployment, financial hardship, low income, and lower educational levels [23]. When comparing our data with the PASOS 2022–2023 study [42], also conducted in Spain among children and adolescents aged 8 to 16 years from the general population, marked differences were observed. In the VG, both boys and girls in our sample showed considerably higher obesity rates than those reported in PASOS, while the prevalence of overweight was slightly lower. In contrast, in the NVG, both overweight and obesity were less prevalent than in the PASOS study cohort.

The findings in our study are in line with earlier data from the THAO intervention study [62], conducted in 2010 by our Research group across multiple Spanish municipalities. According to OF criteria, 15.4% of children were overweight or obese: 9.9% overweight (9.6% boys, 10.1% girls) and 5.5% obese (4.7% boys, 6.3% girls). A later evaluation of the THAO Salud Infantil program (2009–2019) showed a decline in prevalence by 2019: overweight fell to 8.8% and obesity to 3.8%, with higher obesity rates among girls (4.2%) than boys (3.1%). Normal weight significantly increased from 83.4% in 2010 to 86.9% in 2019 (*p* ≤ 0.01). Underweight slightly decreased from 0.8% to 0.6%, with a more marked reduction in boys [54]. In contrast, the present results reveal markedly higher prevalence rates in the vulnerable population, reinforcing the influence of socioeconomic factors.

Further insights can be drawn from school-type stratification in the THAO study, which also analyzed BMI by sex. Among boys, overweight and obesity rates were significantly higher in public schools than in charter or private schools. Among girls, normal weight prevalence was highest in private schools and lowest in public schools, while the inverse pattern was observed for obesity. These findings further reinforce the main conclusions of our study: children from lower socioeconomic backgrounds exhibit higher overweight and obesity rates. Considering that our VG attends public and charter schools and faces social and food insecurity, these outcomes are consistently demonstrated.

Regarding diet quality, the results of our study indicate that low socioeconomic and educational levels are highly associated with poorer diet quality and lower adherence to the MD, in line with previous research [63], and characterized by insufficient consumption of fresh fruits and vegetables, combined with a high intake of low-cost foods that are typically energy-dense and high in fat, sugar, or salt. In the EnKid study (1998–2000) conducted in a population aged between 2 and 24 years [64], 51.5% of participants showed moderate adherence to MD, 44.7% high, and 3.8% low. In our study, moderate adherence was predominant in both groups (vulnerable: 54.3%; non-vulnerable: 51.4%), but low adherence was notably higher among the vulnerable (29.7%), while the non-vulnerable group showed greater high adherence (41.0%). This trend aligns with previous national research [65], which consistently links higher MD adherence with higher educational attainment and socioeconomic status.

These results clearly indicate that lower socioeconomic position is associated not only with poorer adherence to the MD, but also with greater reliance on inexpensive, energy-dense foods, factors that contribute jointly to overweight and obesity in childhood.

In this context, it becomes evident that targeted programs and public policies must urgently focus on economically disadvantaged populations, particularly children and adolescents, to foster the adoption and maintenance of healthy eating habits throughout the life course. Our findings further confirm that dietary patterns in the study population are characterized by high consumption of cereals and sugary foods and low intakes of fruits, vegetables, and fish, consistent with the study data. This imbalance contributes to excessive intake of total fat, saturated fat, cholesterol, sugars, and sodium, alongside significant deficiencies in essential vitamins and minerals. Such patterns deviate substantially from the traditional MD model [62].

Indeed, previous studies have identified children and adolescents as the age groups with the steepest decline in adherence to MD [62]. According to the WHO European Childhood Obesity Surveillance Initiative (COSI), Spain ranks the lowest in the EU for daily vegetable intake among children, and the third lowest for daily fruit intake [52,53]. The persistence of these dietary patterns is particularly concerning, as eating behaviors established in childhood and adolescence often track into adulthood, influencing long-term health outcomes [66].

This stagnation in diet quality may be attributed to multiple factors. Inadequate food intake or poor dietary choices fail to provide the necessary nutrients for a balanced diet. At the individual level, factors such as food preferences, aversions, biological traits (e.g., metabolism or energy requirements), and limited nutritional knowledge hinder informed dietary decision-making. Additionally, broader social determinants, including economic hardship, sedentary behaviours, and low levels of physical activity, further contribute to the problem, as these patterns are often associated with increased consumption of UPF and irregular eating habits, which collectively undermine adherence to healthy dietary models such as the MD [67].

In response to this multifactorial challenge, and given the scientifically established benefits of the MD, national and international institutions have implemented various programs and policies to promote its adoption among children and adolescents. These initiatives aim to foster healthier habits and improve overall well-being both at home and in educational settings [68]. One notable example in Spain is the NAOS Strategy (Nutrition, Physical Activity and Obesity Prevention), launched in 2005 by the Spanish Agency for Food Safety and Nutrition (AESAN). This program seeks to curb the rising prevalence of obesity, particularly in the pediatric population, by encouraging healthier dietary patterns and greater physical activity [69].

In recent years, the rapid increase in UPF consumption has emerged as a major challenge for public health. Consequently, research has also focused on identifying the most harmful UPFs, developing classification systems (still a matter of debate), and proposing prevention strategies to mitigate their health impact [70]. In Spain, the Ministry of Agriculture, Fisheries and Food (MAPA) monitors household food consumption by product group. According to the 2024 Food Consumption Report [71], Spanish households increased their consumption of ready-to-eat meals by 5.1% compared with 2023, reversing the downward trend observed in previous years [72,73]. This category includes canned and frozen meals, soups, pizza, pasta dishes, refrigerated tortillas, and similar products. In parallel, the consumption of bakery and pastry products rose by 2.3%, whereas the intake of chocolate, cocoa, juices, and nectars continued to decline, extending a multi-year trend [72,73]. These results are consistent with our findings, as the vulnerable group in our study showed both the highest UPF intake and the highest prevalence of overweight and obesity, reinforcing the association between social disadvantage, diet quality, and body composition.

The implications of this dietary transition are evident in several longitudinal studies. The PREDIMED study (PREvention with MEDiterranean DIet) in Spain found that higher UPF consumption was associated with significant increases in waist circumference and body weight over time [74]. The ENMIPAD study [75], conducted in the Madrid Region (Spain), confirmed that food insecurity and low socioeconomic status correlate with less healthy lifestyle behaviors in both boys and girls. These findings are consistent with our results: the VG showed both the highest UPF intake and the highest prevalence of overweight and obesity. In our study, children and adolescents from vulnerable households also showed the lowest adherence to the MD, the highest UPF consumption, and the poorest overall diet quality.

To conclude, future, but urgent, strategies to prevent childhood overweight, obesity, and nutrient deficiencies must adopt a comprehensive and holistic approach. Such strategies should not only target individual behaviors but also address broader structural determinants such as food insecurity and socioeconomic inequality in order to maximize their public health impact.

### Strengths and Limitations

The main limitations of this study are its cross-sectional design, which prevents causal interpretation, the relatively small sample size, and the potential recall bias associated with self-reported dietary data. Nonetheless, the inclusion of a socioeconomically diverse pediatric sample, stratified by sex, and the use of both international and national BMI references strengthen the internal validity and contextual interpretation of the results. Additionally, the use of a convenience sampling strategy limits the representativeness of the findings.

## 5. Conclusions

The findings of this study confirm the existence of marked social and nutritional disparities between socioeconomically vulnerable and non-vulnerable children and adolescents in Spain. The differences observed in body composition, food insecurity, and dietary patterns (particularly the lower adherence to the Mediterranean diet and the higher consumption of ultra-processed foods among the vulnerable population) reflect an unequal food environment that fosters the development of overweight, obesity, and other forms of malnutrition from an early age. These disparities not only highlight the impact of social determinants on child health, but also underscore the need for comprehensive interventions that integrate equity-oriented public policies with targeted strategies to promote healthy lifestyles. In this context, it is essential to strengthen school- and community-based programs to improve access to healthy and affordable food, especially among the most disadvantaged groups, in order to break the intergenerational cycle of malnutrition and contribute to the achievement of national and international public health objectives.

## Figures and Tables

**Table 1:** Household food insecurity status among vulnerable and non-vulnerable children and adolescents by sex.

	Vulnerable Population (%) (*n* = 175)	Non-Vulnerable Population (%) (*n* = 105)
TOTAL POPULATION		
Food security	25.1 (*n* = 44)	100.0 (*n* = 105)
Mild food insecurity	22.9 (*n* = 40)	0.0 (*n* = 0)
Moderate food insecurity	23.4 (*n* = 41)	0.0 (*n* = 0)
Severe food insecurity	28.6 (*n* = 50)	0.0 (*n* = 0)
Statistical test	Fisher-Freeman-Halton exact test = 177.048; *p* ≤ 0.001	
BOYS		
Food security	23.1 (*n* = 18)	100.0 (*n* = 64)
Mild food insecurity	14.1 (*n* = 11)	0.0 (*n* = 0)
Moderate food insecurity	26.9 (*n* = 21)	0.0 (*n* = 0)
Severe food insecurity	35.9 (*n* = 28)	0.0 (*n* = 0)
Statistical test	Fisher–Freeman–Halton exact test = 98.698; *p* ≤ 0.001	
GIRLS		
Food security	26.8 (*n* = 26)	100.0 (*n* = 41)
Mild food insecurity	29.9 (*n* = 29)	0.0 (*n* = 0)
Moderate food insecurity	20.6 (*n* = 20)	0.0 (*n* = 0)
Severe food insecurity	22.7 (*n* = 22)	0.0 (*n* = 0)
Statistical test	Fisher–Freeman–Halton exact test = 68.283; *p* ≤ 0.001	

**Table 2:** Anthropometric classification of the total population by vulnerability status and sex.

	Vulnerable Population (*n* = 175)	Non-Vulnerable Population (*n* = 105)
TOTAL POPULATION		
Weight (kg)	39.3 (31.0–49.1)	32.5 *** (27.6–40.2)
Height (cm)	140.5 (130.5–151.0)	137.6 (131.5–148.2)
BMI (kg/m^2^)	19.8 (17.2–22.9)	16.7 *** (15.7–18.0)
Body Fat (%)	27.1 (23.4–32.2)	23.3 *** (20.4–25.3)
BOYS		
Weight (kg)	39.7 (33.1–50.9)	32.5 *** (27.7–37.5)
Height (cm)	143.0 (132.0–151.0)	136.9 (132.0–146.3)
BMI (kg/m^2^)	20.3 (17.5–22.8)	16.8 *** (15.8–17.9)
Body Fat (%)	26.2 (20.9–31.0)	21.0 *** (18.8–24.1)
GIRLS		
Weight (kg)	38.2 (30.2–48.0)	32.7 * (26.2–41.6)
Height (cm)	140.0 (128.5–152.0)	140.3 (129.6–149.5)
BMI (kg/m^2^)	19.4 (16.8–23.1)	16.7 *** (15.4–19.1)
Body Fat (%)	27.8 (24.5–32.9)	24.8 ** (23.6–28.1)

Results are presented as median and interquartile range (IQR). BMI—Body Mass Index, non-percentiled values; not adjusted for age or sex. * *p* ≤ 0.05 compared to the vulnerable population (Mann–Whitney U test). ** *p* ≤ 0.01 compared to the vulnerable population (Mann–Whitney U test). *** *p* ≤ 0.001 compared to the vulnerable population (Mann–Whitney U test).

**Table 3:** Body mass index (BMI) classification by vulnerability status and sex, according to different reference standards.

		Vulnerable Population (%) (*n* = 175)	Non-Vulnerable Population (%) (*n* = 105)
TOTAL POPULATION			
BMI Classification According to OF criteria	Severe thinness	0.0 (*n* = 0)	2.9 (*n* = 3)
	Underweight	4.6 (*n* = 8)	4.8 (*n* = 5)
	Normal weight	51.4 (*n* = 90)	82.9 (*n* = 87)
	Overweight	33.1 (*n* = 58)	9.5 (*n* = 10)
	Obesity	10.9 (*n* = 19)	0.0 (*n* = 0)
Statistical test	Fisher-Freeman-Halton exact test = 45.730; *p* ≤ 0.001		
BMI Classification According to the WHO	Severe thinness	0.0 (*n* = 0)	2.9 (*n* = 3)
	Underweight	4.0 (*n* = 7)	4.8 (*n* = 5)
	Normal weight	43.4 (*n* = 76)	69.5 (*n* = 73)
	Overweight	14.9 (*n* = 26)	16.2 (*n* = 17)
	Obesity	37.7 (*n* = 66)	6.7 (*n* = 7)
Statistical test	Fisher–Freeman–Halton exact test = 41.556; *p* ≤ 0.001		
BOYS			
BMI Classification According to OF criteria	Severe thinness	0.0 (*n* = 0)	0.0 (*n* = 0)
	Underweight	2.6 (*n* = 2)	4.7 (*n* = 3)
	Normal weight	47.4 (*n* = 37)	85.9 (*n* = 55)
	Overweight	41.0 (*n* = 32)	9.4 (*n* = 6)
	Obesity	9.0 (*n* = 7)	0.0 (*n* = 0)
Statistical test	Fisher–Freeman–Halton exact test = 28.664; *p* ≤ 0.001		
BMI Classification According to the WHO	Severe thinness	0.0 (*n* = 0)	1.6 (*n* = 1)
	Underweight	2.6 (*n* = 2)	4.7 (*n* = 3)
	Normal weight	41.0 (*n* = 32)	68.8 (*n* = 44)
	Overweight	14.1 (*n* = 11)	18.8 (*n* = 12)
	Obesity	42.3 (*n* = 33)	6.3 (*n* = 4)
Statistical test	Fisher–Freeman–Halton exact test = 26.845; *p* ≤ 0.001		
GIRLS			
BMI Classification According to OF criteria	Severe thinness	0.0 (*n* = 0)	7.3 (*n* = 3)
	Underweight	6.2 (*n* = 6)	4.9 (*n* = 2)
	Normal weight	54.6 (*n* = 53)	78.0 (*n* = 32)
	Overweight	26.8 (*n* = 26)	9.8 (*n* = 4)
	Obesity	12.4 (*n* = 12)	0.0 (*n* = 0)
Statistical test	Fisher–Freeman–Halton exact test = 18.189; *p* ≤ 0.001		
BMI Classification According to the WHO	Severe thinness	0.0 (*n* = 0)	4.9 (*n* = 2)
	Underweight	5.2 (*n* = 5)	4.9 (*n* = 2)
	Normal weight	45.4 (*n* = 44)	70.7 (*n* = 29)
	Overweight	15.5 (*n* = 15)	12.2 (*n* = 5)
	Obesity	34.0 (*n* = 33)	7.3 (*n* = 3)
Statistical test	Fisher–Freeman–Halton exact test = 16.521; *p* ≤ 0.001		

BMI—Body Mass Index, OF—Orbegozo Foundation; WHO—World Health Organization.

**Table 4:** Dietary quality indices in vulnerable and non-vulnerable populations, stratified by sex.

	Vulnerable Population (%) (*n* = 175)	Non-Vulnerable Population (%) (*n* = 105)
HEALTHY EATING INDEX.		
Total population		
Unhealthy	29.6 (*n* = 42)	4.8 (*n* = 5)
Needs improvement	70.4 (*n* = 100)	90.5 (*n* = 95)
Healthy	0.0 (*n* = 0)	4.8 (*n* = 5)
Statistical test	Fisher–Freeman–Halton exact test = 29.372; *p* ≤ 0.001	
Boys		
Unhealthy	31.7 (*n* = 19)	6.3 (*n* = 4)
Needs improvement	68.3 (*n* = 41)	87.5 (*n* = 56)
Healthy	0.0 (*n* = 0)	6.3 (*n* = 4)
Statistical test	Fisher–Freeman–Halton exact test = 16.008; *p* ≤ 0.001	
Girls		
Unhealthy	28.0 (*n* = 23)	2.4 (*n* = 1)
Needs improvement	72.0 (*n* = 59)	95.1 (*n* = 39)
Healthy	0.0 (*n* = 0)	2.4 (*n* = 1)
Statistical test	Fisher–Freeman–Halton exact test = 14.710; *p* ≤ 0.001	
ADHERENCE TO THE MEDITERRANEAN DIET (MD)		
Total population		
Low	29.7 (*n* = 52)	7.6 (*n* = 8)
Moderate	54.3 (*n* = 95)	51.4 (*n* = 54)
High	16.0 (*n* = 28)	41.0 (*n* = 43)
Statistical test	Fisher–Freeman–Halton exact test = 32.320; *p* ≤ 0.001.	
Boys		
Low	32.1 (*n* = 25)	7.8 (*n* = 5)
Moderate	55.1 (*n* = 43)	51.6 (*n* = 33)
High	12.8 (*n* = 10)	40.6 (*n* = 26)
Statistical test	Fisher–Freeman–Halton exact test = 21.118; *p* ≤ 0.001	
Girls		
Low	27.8 (*n* = 27)	7.3 (*n* = 3)
Moderate	53.6 (*n* = 52)	51.2 (*n* = 21)
High	18.6 (*n* = 18)	41.5 (*n* = 17)
Statistical test	Fisher–Freeman–Halton exact test = 11.704; *p* ≤ 0.001	
CONSUMPTION OF ULTRA-PROCESSED FOODS (UPF)		
Total population		
Low	10.3 (*n* = 18)	29.5 (*n* = 31)
Medium	80.6 (*n* = 141)	69.5 (*n* = 73)
High	9.1 (*n* = 16)	1.0 (*n* = 1)
Statistical test	Fisher–Freeman–Halton exact test = 22.432; *p* ≤ 0.001	
Boys		
Low	6.4 (*n* = 5)	28.1 (*n* = 18)
Medium	83.3 (*n* = 65)	70.3 (*n* = 45)
High	10.3 (*n* = 8)	1.6 (*n* = 1)
Statistical test	Fisher–Freeman–Halton exact test = 15.155; *p* ≤ 0.001	
Girls		
Low	13.4 (*n* = 13)	31.7 (*n* = 13)
Medium	78.4 (*n* = 76)	68.3 (*n* = 28)
High	8.2 (*n* = 8)	0.0 (*n* = 0)
Statistical test	Fisher–Freeman–Halton exact test = 8.504; *p* ≤ 0.001	

**Table 5:** Determinants of vulnerability in the study population.

Variables	Categories	ODDS RATIO	95% CI %
Sex	Boys	ref	
	Girls	0.374 **	[0.203–0.692]
Age		0.954	[0.818–1.114]
BMI		1.410 ***	[1.250–1.592]
Adherence to MD	Low	ref	
	Moderate	3.178 ***	[1602–6307]
	High	10.267 ***	[3756–28,063]
UPF intake	Low	ref	
	Medium	3.178 **	[1.441–7.102]
	High	24.570 **	[2.613–231.012]

MD—Mediterranean Diet; UPF—Ultra-Processed Foods; BMI—Body Mass Index, 95% CI: 95% Confidence Interval. Asterisks indicate statistical significance levels: ** *p* < 0.01; *** *p* < 0.001. *n* = 280. R^2^ Cox and Snell = 0.317. R^2^ Nagelkerke = 0.433. Prob X^2^ < 0.001.

## Data Availability

The data presented in this study are available upon reasonable request from the corresponding author. The data are not publicly available due to ethical reasons.

## Abbreviations

The following abbreviations are used in this manuscript:

VG	Vulnerable group
NVG	Non-vulnerable group
MD	Mediterranean Diet
BMI	Body Mass Index
SOFI	State of Food Security and Nutrition in the World
UPF	Ultra-processed Foods
NGO	Non-Governmental Organization
HFIAS	Household Food Insecurity Access
FANTA	Food and Nutrition Technical Assistance
HPF	High Ultra-Processed Food
ISAK	International Standards for Anthropometric Assessment
OF	Faustino Orbegozo
WHO	World Health Organization
SD	Standard Deviations
IQR	Interquartile Ranges
OR	Odds ratios
CI	Confidence Intervals
INE	National Institute of Statistics
FIES	Food Insecurity Experience Scale
EU	European Union
COSI	Childhood Obesity Surveillance Initiative
NAOS	Nutrition, Physical Activity and Obesity Prevention
AESAN	Agency for Food Safety and Nutrition
MAPA	Ministry of Agriculture, Fisheries and Food
PREDIMED	Prevention with Mediterranean Diet
